# The risk of pediatric cardiovascular diseases in offspring born to mothers with systemic lupus erythematosus: a nationwide study

**DOI:** 10.3389/fped.2023.1294823

**Published:** 2023-12-05

**Authors:** Jong Ho Cha, Jae Kyoon Hwang, Young-Jin Choi, Jae Yoon Na

**Affiliations:** ^1^ Department of Pediatrics, Hanyang University Hospital, Seoul, Republic of Korea; ^2^ Department of Pediatrics, Hanyang University Guri Hospital, Guri, Republic of Korea; ^3^ Department of Pediatrics, Hanyang University College of Medicine, Seoul, Republic of Korea

**Keywords:** systematic lupus erythematosus, nationwide study, congenital heart disease, neonatal lupus erythematosus, mucocutaneous lymph node syndrome

## Abstract

**Background:**

Systemic lupus erythematosus (SLE), a common autoimmune disease predominantly affecting women, has been linked to various complications during pregnancy. The transfer of anti-Ro/SSA antibodies from SLE-affected mothers to their offspring can lead to neonatal lupus and cardiac issues. This study investigated the association between maternal SLE and the risk of pediatric cardiovascular disorders.

**Methods:**

The study utilized South Korea's National Health Insurance Service (NHIS) database, covering 3,505,737 children born between 2007 and 2017 and tracked until 2020. Maternal SLE cases were identified using the World Health Organization's International Classification of Diseases Tenth revision (ICD-10) codes and linked with delivery records. Cardiologic disorders were categorized into congenital heart disease (CHD), arrhythmic disorders, and acquired heart disease. Propensity score matching with 1:4 ratios was applied to the set control group.

**Results:**

Among 3,505,737 children, 0.7% (*n* = 23,330) were born to mothers with SLE. The incidence of preterm birth was significantly higher in the maternal SLE group (5.9% vs. 3.0%). Compared with the control group, children born to mothers with SLE exhibited a significantly elevated risk of overall CHDs (5.5%, adjusted odds ratio [aOR] 1.21; 95% confidence interval [CI] 1.14–1.29), including atrial septal defect (1.18; 1.09–1.28) and patent ductus arteriosus (1.15; 1.03–1.30). In addition, a notably higher risk was observed in arrhythmic disorders (complete atrioventricular block 7.20; 2.41–21.49) and acquired cardiac disorders, including cardiomyopathy (1.40; 1.17–1.68) and mucocutaneous lymph node syndrome (MCLS) (1.27; 1.15–1.43).

**Conclusions:**

Maternal SLE is associated with congenital and acquired cardiac disorders in offspring, including structural, arrhythmic, and MCLS. This study highlights the need for continuous cardiovascular monitoring from the prenatal stage to preadolescence in these children due to multifactorial influences involving maternal autoantibodies, genetic predisposition, and environmental factors.

## 1. Introduction

Systematic lupus erythematosus (SLE) is one of the most common autoimmune diseases that affect multi-system abnormalities (1). It predominantly affects women, with up to a 9-fold difference in incidence during reproductive ages (2). Previous studies demonstrated that SLE causes a variety of complications in mothers, such as pre-eclampsia, preterm birth, and fetal demise (3). Although fetal loss has been improved due to clinical protocols and prenatal monitoring, it is still an important issue in SLE, and health surveillance of the surviving infants should be maintained (4).

The passive transfer of anti-Ro/SSA autoantibodies from an SLE-affected mother to her offspring is known to increase several clinical conditions, such as neonatal lupus rash and hepatic and central nervous system involvement (5, 6). Canadian (7) and Chinese (8) studies have shown that offspring born from mothers with SLE were more likely to have adverse neonatal outcomes, such as neonatal intensive care unit admission, preterm birth, and patent ductus arteriosus (PDA). In particular, cardiac findings are notable, and the most serious complication is third-degree atrioventricular (AV) block, affecting approximately 2% of exposed pregnancies (9). Cutaneous and hematologic abnormalities are usually known to subside by 6–8 months of age with the resolution of maternal antibodies (10, 11). Conversely, it is known that neonatal SLE is related to various cardiovascular abnormalities, including arrhythmias, structural abnormalities, and cardiomyopathies (12). Given that these adverse conditions could result in permanent damage, the risk of cardiovascular abnormalities should be assessed (13).

Until now, previous studies regarding pregnancy-induced outcomes of SLE have been confined to maternal perspectives (14, 15) or addressed adverse outcomes in the neonatal period with a relatively small sample size of children (3, 7, 8). Thus, the cardiovascular outcomes of offspring born to mothers with SLE should be investigated. Herein, using nationwide health claims data from 2007 to 2017, we evaluated the association of maternal SLE with pediatric cardiovascular disorders, including congenital and acquired disorders.

## 2. Methods

### 2.1. Data source and study population

We obtained data on children born between 2007 and 2017 and followed their medical claims data until 2020 in the National Health Insurance Service (NHIS) database. In South Korea, more than 97% of the population are registered and covered by national medical insurance. The NHIS collects medical claims data and has made them available to use for research purposes since 2007. Diagnoses data are registered with the World Health Organization's International Classification of Diseases Tenth revision (ICD-10).

We extracted prenatal records by linking the data of the individuals (children) with the data of their respective mothers. For the extraction, we only linked mothers when a delivery record was registered in the NHIS database and the date of delivery was defined as the date of the birth. Maternal SLE was defined as an individual who visited an inpatient or outpatient clinic on more than one occasion and was assigned the ICD-10 code “M32” prior to delivery. We included 3,505,737 children registered in the NHIS database in the study. We excluded 138,549 individuals without baseline demographic information. Among the population, 23,330 children were born to mothers diagnosed with SLE. The study was approved by the Institutional Review Board of Hanyang University Guri Hospital (IRB No. GURI 2023-02-026). The requirement for informed consent was waived because public data from the NHIS were used (NHIS-2023-1-396). The reporting of this study conforms to the Strengthening the Reporting of Observational Studies in Epidemiology (STROBE) guidelines (online Supplementary Table 1).

### 2.2. Cardiologic manifestations and clinical variables

We categorized pediatric cardiologic disorders as congenital heart disease (CHD), arrhythmic disorders, and other acquired cardiologic disorders [e.g., cardiomyopathy (CMP) and mucocutaneous lymph node syndrome (MCLS)]. Moreover, we investigated neonatal lupus and death as outcomes. Each outcome was defined as more than two outpatient or inpatient visits with respective ICD-10 diagnostic codes.

As maternal characteristics, we selected cesarean section, intrauterine growth retardation (IUGR), pregnancy induced hypertension (PIH), gestational diabetes mellitus, chronic kidney disorders, and maternal CHD as variables of interests. Maternal CHD was defined as a mother who was assigned a diagnostic CHD code (Q2) during the observation period. Growing evidence suggests that genetic predisposition plays a crucial role in the pathogenesis of CHD; therefore, maternal CHD should be taken into account as a matching variable (16).

### 2.3. Statistical analysis

The demographic characteristics of the participants were calculated with weighted percentages and compared with the standardized mean difference (SMD). The propensity score was calculated using variables including birth year, sex, socioeconomic status, residence, preterm birth, and maternal CHDs. Additionally, differences in maternal characteristics and cardiac manifestations between two groups were compared using a chi-square test and featured with weighted percentages and a *p* value. Although SLE increasing the risk of preterm birth was previously known, we included preterm birth in the propensity score variables as it significantly increases the risk of CHD (15). The nearest available matching based on the estimated propensity score was performed with 1:4 ratios. Then, logistic regression analysis was used to estimate the odds ratio (OR) with a 95% confidence interval (CI) of CHD risk of children with maternal SLE. Statistical significance was determined using two-sided tests, with a *p* value of less than 0.05 considered significant. Statistical analyses were performed using SAS version 9.4 (SAS Institute Inc., Cary, NC, USA) and R version 4.2.1 (www.R-project.org).

## 3. Results

**Figure 1:**
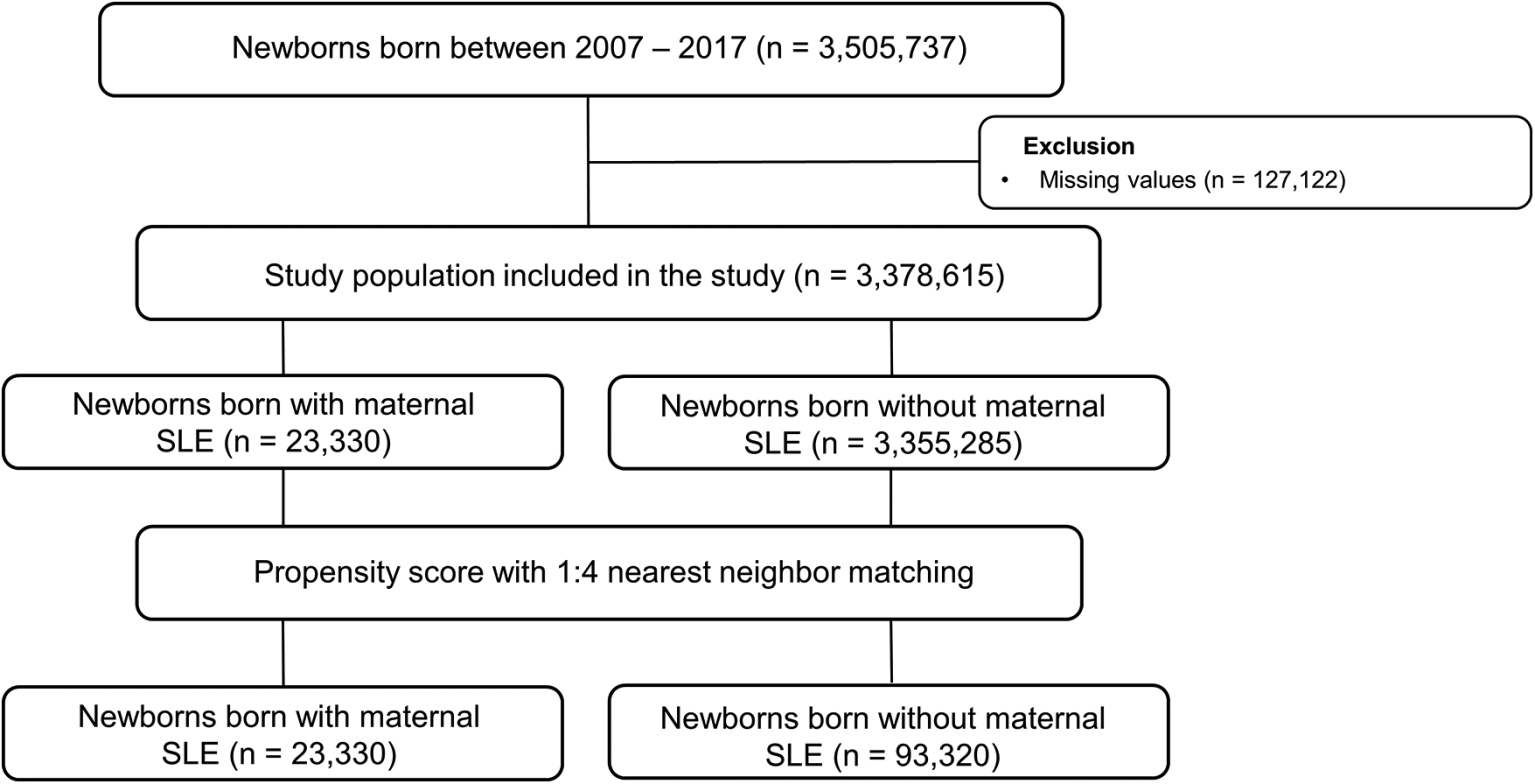
Selection flow of the population included in the analysis. SLE, systematic lupus erythematosus.

We included 3,505,737 children registered in the national health insurance database in the study (Figure 1). We excluded 127,122 individuals without baseline demographic information. Among the population, 23,330 children were born from mothers diagnosed with SLE. The baseline demographic findings of the study population are shown in Table 1. Before matching, offspring born from mothers with SLE had a significantly higher incidence of preterm birth and maternal congenital heart disease (SMD > 0.1). After propensity score matching, the SMD between children with and without maternal SLE was lower than 0.1 in all variables, such as birth year, sex, socioeconomic status, residence, preterm birth, and maternal CHDs. The incidences of preterm birth and maternal CHD were 5.9% and 13.4%, respectively.

**Table 1:** Baseline characteristics of the pediatric population included in the study before and after propensity score matching.

	Children without maternal SLE (before matching) (*N* = 3,355,285)	Children without maternal SLE (after matching) (*N* = 93,320)	Children with maternal SLE (*N* = 23,330)	SMD (before matching)	SMD^c^ (after matching)
Birth year				0.08	<0.001
2007–2010	816,412 (26.4)	24,776 (29.1)	6,194 (29.1)		
2011–2014	1,369,433 (44.3)	37,972 (44.5)	9,493 (44.5)		
2015–2017	906,788 (29.3)	22,504 (26.4)	5,626 (26.4)		
Sex				<0.01	<0.01
Boys	1,719,456 (51.2)	47,993 (51.4)	12,019 (51.5)		
Girls	1,635,829 (48.8)	45,327 (48.6)	11,311 (48.5)		
SES^a^				0.07	<0.001
Q1 (lowest)	356,999 (10.6)	9,551 (10.2)	2,387 (10.2)		
Q2	755,787 (22.5)	19,340 (20.7)	4,825 (20.7)		
Q3	1,349,712 (40.2)	36,857 (39.5)	9,225 (39.5)		
Q4 (highest)	892,787 (26.6)	27,572 (29.5)	6,893 (29.5)		
Residence^b^				0.14	<0.001
City	1,498,983 (44.7)	48,260 (51.7)	12,065 (51.7)		
Rural	1,856,302 (55.3)	45,060 (48.3)	11,265 (48.3)		
Preterm birth	101,472 (3.0)	5,544 (5.9)	1,386 (5.9)	0.14	<0.001
Maternal CHD	218,812 (6.5)	12,496 (13.4)	3,124 (13.4)	0.23	0.02

Data are expressed as numbers (weighted %).

SLE, systematic lupus erythematosus; SMD, standardized mean difference; SES, socioeconomic status; Q, quartile; CHD, congenital heart disease.

^a^ Based on the amount of health insurance premiums. Income status was categorized into quartiles.

^b^ City area was defined as Seoul and six major metropolitan cities (Busan, Incheon, Gwangju, Daegu, Daejeon, and Ulsan).

^c^ Propensity score matching with birth year, sex, SES, residence, preterm birth, and maternal CHD.

Table 2 summarizes the comparisons between maternal and cardiologic manifestations between two groups. For maternal characteristics, mothers with SLE had a higher incidence of cesarean section, IUGR, PIH, and chronic kidney disorders. However, these differences were not statistically significant.

**Table 2:** Prenatal and obstetric characteristics of the population included in the study.

	Children without maternal SLE (before matching) (*N* = 3,355,285)		Children without maternal SLE (after matching) (*N* = 93,320)		Children with maternal SLE (*N* = 23,330)		SMD (before matching)	SMD^a^ (after matching)
	*N*	Weighted %	*N*	Weighted %	*N*	Weighted %		
Maternal characteristics								
Cesarean section	1,220,053	36.4	34,125	36.6	9,228	39.6	0.07	0.06
IUGR	107,949	3.2	3,362	3.6	1,088	4.7	0.07	0.05
PIH	49,271	1.5	1,802	1.9	749	3.2	0.12	0.08
Gestational diabetes mellitus	1,377,325	41.0	37,792	40.5	9,206	39.5	0.03	0.02
Chronic kidney disorders	12,793	0.4	1,802	1.9	557	2.4	0.17	0.16

Data are expressed as numbers (weighted %).

SLE, systematic lupus erythematosus; SMD, standardized mean difference; IUGR, intrauterine growth retardation; PIH, pregnancy induced hypertension.

^a^ Propensity score matching with birth year, sex, SES, residence, preterm birth, and maternal CHD.

For CHD, children of mothers with SLE had a significantly higher incidence of ventricular septal defect (VSD), atrial septal defect (ASD), PDA, and all CHDs. For arrhythmic disorders, 15 cases of AV block were identified in children of mothers with SLE, which was significantly higher than in the control group (six cases, *p* < 0.05). Additionally, the incidence of other arrhythmias was higher than in the control group (0.7% vs. 0.5%). However, the differences in the incidences of cardiac arrest, other block, atrial flutter, and tachyarrhythmia were not significant. The incidences of neonatal lupus and childhood mortality in the SLE mother group were 0.5% and 0.2%, respectively, which were higher than those in the control group. A description of the number of cases of CHD in children of mothers with SLE is summarized in Supplementary Table 2.

**Figure 2:**
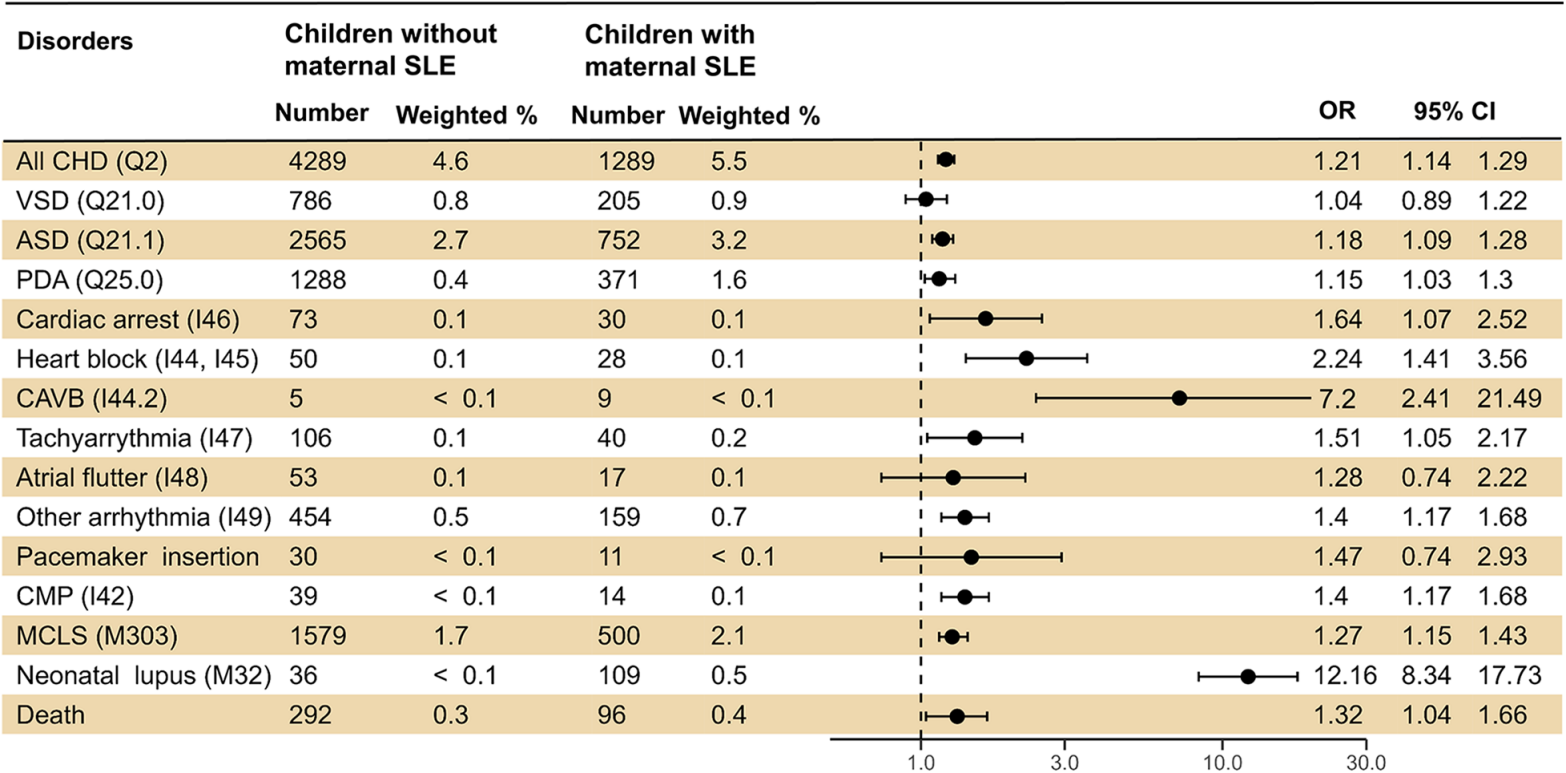
Logistic regression analysis presenting the risk of cardiovascular diseases in offspring born to mothers with SLE. SLE, systematic lupus erythematosus; OR, odds ratio; CI, confidence interval; CHD, congenital heart disease; VSD, ventricular septal defect; ASD, atrial septal defect; PDA, patent ductus arteriosus; AV, atrioventricular; CMP, cardiomyopathy; MCLS, mucocutaneous lymph node syndrome.

Children of mothers with SLE had a significantly higher risk of overall CHD (OR 1.23; 95% CI 1.15–1.31), as well as VSD, ASD, and PDA. Additionally, the risk of AV block was the highest [10.01 (3.88–25.79)], followed by other arrhythmias [1.37 (1.14–1.64)]. For acquired cardiac disorders, there was a higher risk of CMP [12.16 (8.34–17.73)] and MCLS [1.29 (1.17–1.43)]. Finally, the enhanced risk of neonatal lupus [12.16 (8.34–17.73)] and pediatric mortality [1.48 (1.17–1.87)] was also investigated (Figure 2).

## 4. Discussion

This nationwide study demonstrated that offspring born from mothers with SLE had a significantly higher risk of congenital and acquired pediatric cardiologic disorders. In detail, maternal SLE was associated with congenital structural heart disorders. By contrast, the association with arrhythmic disorders was confined to AV block. Additionally, maternal SLE was associated with elevated risks of MCLS, CMP, and pediatric mortality.

To our knowledge, this is the first study to use nationwide data to assess the risk of cardiovascular disorders in offspring of mothers with SLE. It is already well-documented that SLE is strongly associated with pregnancy outcomes, and in our study, SLE was associated with the incidence of complications such as cesarean section, IUGR, PIH, and preterm birth. We did not include maternal comorbidities in the propensity score variables, except for maternal CHDs, as the association of these variables with pediatric cardiovascular disorder is unclear (17).

The incidence of overall CHD in offspring of SLE mothers was 5.5%, which was higher than previous reports (18, 19). There was an increased risk of preterm birth in the maternal SLE group, which can be interpreted as a key factor for the increased risk of CHD. However, according to our risk, the risk of CHD was still evident even after controlling for preterm birth. Further studies investigating whether SLE autoantibodies contribute to the pathogenesis of CHD should be undertaken. Rather, it could be the overdiagnosis bias, given that mothers with SLE are more likely to undergo a preemptive cardiologic work up during pregnancy. In addition, children born from mothers diagnosed with SLE may have been overdiagnosed with CHDs due to repeated echocardiograms, such as patent foramen ovale/ASD and PDA that can resolve spontaneously later in life.

Among arrhythmic disorders, we identified nine cases of third-degree AV block, with an estimated incidence of 1 per 5,000 live births, which was higher than in a previous study (20). The risk of third-degree AV block was 10-fold higher in the SLE mother group. Although we could not investigate the status of maternal autoantibodies due to methodological limitations, our results support the notion that the majority of pediatric third-degree AV block cases are due to maternal autoantibodies (21). We found that offspring born to mothers with SLE also have a prominent risk of first- and second-grade AV block. The initial manifestation of AV block could be either first or second degree, and those whose features usually spontaneously resolve within the first month of life (22, 23). This implies that the inflammatory reaction caused by maternal autoantibodies is mild and reversible (24). Neonatal lupus erythematosus is known to be closely associated with a variety of arrhythmic disorders (13). Our results showed that these adverse effects were limited to AV block, premature beat, ectopic beats, sinus arrhythmia, and long QT disorders.

Notably, we underline that the offspring born from SLE mothers had significantly higher risk of CMP and MCLS. According to previous studies, 15%–20% of affected children with third degree AV block could develop into more prominent myocardial disease, progressing to CMP (21, 25). Given that the incidence of CMP was higher than that of AV block, we assume that additional mechanism could underlie. The mechanism of cardiac damage resulting from maternal autoantibodies is still unclear, but the most known hypothesis is that maternal autoantibodies interfere with macrophage-mediated clearance of apoptotic cardiocytes by releasing pro-inflammatory cytokines and inducing inflammatory reactions, with subsequent cardiac inflammation and scarring (26–28). Nevertheless, we could not ascertain that maternal autoantibodies solely affected the pathogenesis of the CMP since pediatric CMP could result from many underlying cardiovascular conditions, not only maternal rheumatic disorders, but from coronary artery abnormalities, tachyarrhythmia, and secondary to underlying conditions (29).

There have been two studies with large sample sizes regarding the impact of maternal autoimmune disorders on MCLS, with conflicting results. Chu et al. (30) investigated 7,178 children and showed that SLE did not have an adverse effect on MCLS. However, when a broader range of autoimmune diseases, such as Sjogren's and rheumatoid arthritis, were included, maternal immune diseases were significantly associated. Meanwhile, a cohort study in Canada, with a follow-up for 12 years, stated that maternal rheumatic disorders had a 1.84-fold increase in MCLS in offspring; in particular, a higher risk was expected until 5 years of age (OR: 2.06, 95% CI: 1.77–2.41) (31). Unlike previous studies, we only included children who were born to mothers diagnosed with SLE, and considered maternal CHDs in the variables. It is known that transplacental autoantibodies disappear within the first 12 months. Thus, an inflammatory cascade known to be the pathogenesis of arrhythmic disorders would not explain the relationship with MCLS.

MCLS is a systemic vasculitis that affects small- and medium-sized arteries with elevated cytokine levels (32). Growing evidence suggests that immune complexes play a crucial role in the pathogenesis of MCLS (33). In addition, according to a genome-wide association study, autoimmune disease (e.g., SLE and rheumatoid arthritis) and MCLS share common candidate genes (e.g., BLK and CD40) (34). Although we could not verify the causality of the relationship, the genetic predisposition could affect the pathogenesis of MCLS, which would require clinical attention in febrile or infectious cases.

The strength of our study lies in the large sample size of nationwide health claims data, presenting pediatric cardiovascular diseases of offspring born to mothers with SLE. In contrast to previous studies that focused on the prenatal and neonatal health outcomes of childhood, we investigated overall pediatric cardiovascular disorders up until the pre-adolescent period. However, this study has several limitations due to methodological issues. First, we could not investigate the clinical information (e.g., maternal age, SLE flare, onset of maternal SLE, and duration of SLE) and laboratory findings (e.g., antibody status) of mothers. Such clinical symptoms and laboratory findings could not be obtained from our database, which is based on ICD-10 codes. The autoantibody profile is highly variable in SLE groups and could present different clinical manifestations of offspring (24). Approximately one half of pregnant SLE patients experience flare ups and should be considered a serious risk factor. Additionally, genetic predisposition to CHD was not identifiable due to the characteristics of our data. Further studies based on the nationwide data with genetic information should be carried out. Second, for children, we could not investigate the echocardiography and echocardiogram findings for the diagnosis of cardiovascular disorders. Although we defined outcomes using diagnoses confirmed by pediatricians, the objective clinical findings would be helpful in assessing cardiovascular outcomes in children. Finally, as we obtained maternal data by linking ICD-10 codes regarding delivery, we could not identify abortion and stillbirth cases of mothers with SLE. To evaluate the CHD risk, results from fetal echocardiography should be investigated in further studies.

In conclusion, using nationwide registry health claims data, this study investigated the risk of pediatric cardiovascular disorders in offspring born to mothers with SLE. In addition to the significant risk of complete AV block, maternal SLE was significantly associated with overall CHD, CMP, acquired cardiovascular disorders, such as MCLS, and even mortality. We suspect these associations to be multifactorial etiology, with the combined effects of maternal autoantibodies, genetic predisposition, and environmental factors, which needs to be confirmed in further studies. We underline that offspring born to mothers with SLE should undergo regular surveillance of their cardiovascular systems from the prenatal period through to pre-adolescence.

## Data Availability

The raw data supporting the conclusions of this article will be made available by the authors, without undue reservation.

## References

[1] Yu KH See LC Kuo CF Chou IJ Chou MJ. Prevalence and incidence in patients with autoimmune rheumatic diseases: a nationwide population-based study in Taiwan. Arthritis Care Res (Hoboken). (2013) 65:244–50. 10.1002/acr.21820 22899470

[2] Weckerle CE Niewold TB. The unexplained female predominance of systemic lupus erythematosus: clues from genetic and cytokine studies. Clin Rev Allergy Immunol. (2011) 40:42–9. 10.1007/s12016-009-8192-4 20063186 PMC2891868

[3] Bundhun PK Soogund MZS Huang F. Impact of systemic lupus erythematosus on maternal and fetal outcomes following pregnancy: a meta-analysis of studies published between years 2001–2016. J Autoimmun. (2017) 79:17–27. 10.1016/j.jaut.2017.02.009 28256367

[4] Clark CA Spitzer KA Laskin CA. Decrease in pregnancy loss rates in patients with systemic lupus erythematosus over a 40-year period. J Rheumatol. (2005) 32:1709–12. 16142865

[5] Brucato A Cimaz R Caporali R Ramoni V Buyon J. Pregnancy outcomes in patients with autoimmune diseases and anti-ro/SSA antibodies. Clin Rev Allergy Immunol. (2011) 40:27–41. 10.1007/s12016-009-8190-6 20012231 PMC3558034

[6] Izmirly PM Saxena A Kim MY Wang D Sahl SK Llanos C Maternal and fetal factors associated with mortality and morbidity in a multi–racial/ethnic registry of anti-SSA/ro–associated cardiac neonatal lupus. Circulation. (2011) 124:1927–35. 10.1161/CIRCULATIONAHA.111.033894 21969015 PMC3206147

[7] Nili F McLeod L O’Connell C Sutton E McMillan D. Maternal and neonatal outcomes in pregnancies complicated by systemic lupus erythematosus: a population-based study. J Obstet Gynaecol Can. (2013) 35:323–8. 10.1016/S1701-2163(15)30959-2 23660039

[8] Wu J Ma J Bao C Di W Zhang W-H. Pregnancy outcomes among Chinese women with and without systemic lupus erythematosus: a retrospective cohort study. BMJ Open. (2018) 8:e020909. 10.1136/bmjopen-2017-020909 29654043 PMC5905749

[9] Lateef A Petri M. Systemic lupus erythematosus and pregnancy. Rheum Dis Clin. (2017) 43:215–26. 10.1016/j.rdc.2016.12.009 28390564

[10] Prendiville JS Cabral DA Poskitt KJ Au S Sargent MA. Central nervous system involvement in neonatal lupus erythematosus. Pediatr Dermatol. (2003) 20:60–7. 10.1046/j.1525-1470.2003.03014.x 12558850

[11] Zuppa AA Riccardi R Frezza S Gallini F Luciano RMP Alighieri G Neonatal lupus: follow-up in infants with anti-SSA/ro antibodies and review of the literature. Autoimmun Rev. (2017) 16:427–32. 10.1016/j.autrev.2017.02.010 28212920

[12] Vanoni F Lava SA Fossali EF Cavalli R Simonetti GD Bianchetti MG Neonatal systemic lupus erythematosus syndrome: a comprehensive review. Clin Rev Allergy Immunol. (2017) 53:469–76. 10.1007/s12016-017-8653-0 29116459

[13] Hornberger L Al Rajaa N. Spectrum of cardiac involvement in neonatal lupus. Scand J Immunol. (2010) 72:189–97. 10.1111/j.1365-3083.2010.02437.x 20696015

[14] Joo YB Kim K-J Park K-S Park Y-J. Pregnancy rates and perinatal outcomes in women with systemic lupus erythematosus: data from the Korean national health claims database. Clin Rheumatol. (2021) 40:2243–50. 10.1007/s10067-020-05496-4 33184707

[15] Park J-S Chung MK Lim H Lee J Lee CH. Risk of pregnancy complications and low birth weight offsprings in Korean women with rheumatic diseases: a nationwide population-based study. J Korean Med Sci. (2022) 37(2):e18. 10.3346/jkms.2022.37.e18 35014229 PMC8748664

[16] Zaidi S Brueckner M. Genetics and genomics of congenital heart disease. Circ Res. (2017) 120:923–40. 10.1161/CIRCRESAHA.116.309140 28302740 PMC5557504

[17] Leybovitz-Haleluya N Wainstock T Landau D Sheiner E. Maternal gestational diabetes mellitus and the risk of subsequent pediatric cardiovascular diseases of the offspring: a population-based cohort study with up to 18 years of follow up. Acta Diabetol. (2018) 55:1037–42. 10.1007/s00592-018-1176-1 29936651

[18] Costedoat-Chalumeau N Amoura Z Lupoglazoff JM Thi Huong DL Denjoy I Vauthier D Outcome of pregnancies in patients with anti-SSA/ro antibodies: a study of 165 pregnancies, with special focus on electrocardiographic variations in the children and comparison with a control group. Arthritis Rheum. (2004) 50:3187–94. 10.1002/art.20554 15476223

[19] Solomon DG. Birth order, gender and recurrence rate in autoantibody-associated congenital heart block: implications for pathogenesis and family counseling. Lupus. (2003) 12:646. 10.1191/0961203303lu425xx 12945727

[20] Cioffi GM Gasperetti A Tersalvi G Schiavone M Compagnucci P Sozzi FB Etiology and device therapy in complete atrioventricular block in pediatric and young adult population: contemporary review and new perspectives. J Cardiovasc Electrophysiol. (2021) 32:3082–94. 10.1111/jce.15255 34570400

[21] Jaeggi ET Hamilton RM Silverman ED Zamora SA Hornberger LK. Outcome of children with fetal, neonatal or childhood diagnosis of isolated congenital atrioventricular block: a single institution’s experience of 30 years. J Am Coll Cardiol. (2002) 39:130–7. 10.1016/S0735-1097(01)01697-7 11755298

[22] Bergman G Wahren-Herlenius M Sonesson SE. Diagnostic precision of Doppler flow echocardiography in fetuses at risk for atrioventricular block. Ultrasound Obstet Gynecol. (2010) 36:561–6. 10.1002/uog.7532 20069676

[23] Rein A Mevorach D Perles Z Gavri S Nadjari M Nir A Early diagnosis and treatment of atrioventricular block in the fetus exposed to maternal anti-SSA/Ro-SSB/La antibodies: a prospective, observational, fetal kinetocardiogram–based study. Circulation. (2009) 119:1867–72. 10.1161/CIRCULATIONAHA.108.773143 19332471

[24] Salomonsson S Dzikaite V Zeffer E Eliasson H Ambrosi A Bergman G A population-based investigation of the autoantibody profile in mothers of children with atrioventricular block. Scand J Immunol. (2011) 74:511–7. 10.1111/j.1365-3083.2011.02610.x 21815910

[25] Moak JP Barron KS Hougen TJ Wiles HB Balaji S Sreeram N Congenital heart block: development of late-onset cardiomyopathy, a previously underappreciated sequela. J Am Coll Cardiol. (2001) 37:238–42. 10.1016/S0735-1097(00)01048-2 11153745

[26] Buyon JP Clancy RM Friedman DM. Cardiac manifestations of neonatal lupus erythematosus: guidelines to management, integrating clues from the bench and bedside. Nat Rev Rheumatol. (2009) 5:139–48. 10.1038/ncprheum1018 19252519

[27] Izmirly PM Kim MY Llanos C Le PU Guerra MM Askanase AD Evaluation of the risk of anti-SSA/Ro-SSB/La antibody-associated cardiac manifestations of neonatal lupus in fetuses of mothers with systemic lupus erythematosus exposed to hydroxychloroquine. Ann Rheum Dis. (2010) 69:1827–30. 10.1136/ard.2009.119263 20447951 PMC3593727

[28] Clancy RM Alvarez D Komissarova E Barrat FJ Swartz J Buyon JP. Ro60-associated single-stranded RNA links inflammation with fetal cardiac fibrosis via ligation of TLRs: a novel pathway to autoimmune-associated heart block. J Immunol. (2010) 184:2148–55. 10.4049/jimmunol.0902248 20089705 PMC3551297

[29] Lee TM Hsu DT Kantor P Towbin JA Ware SM Colan SD Pediatric cardiomyopathies. Circ Res. (2017) 121:855–73. 10.1161/CIRCRESAHA.116.309386 28912187 PMC5657298

[30] Chu H-W Lin C-H Lin M-C Hsu Y-C. Increased risk of Kawasaki disease in infants born of mothers with immune disorders. Front Pediatr. (2021) 9:659598. 10.3389/fped.2021.659598 34055693 PMC8160226

[31] Belkaibech S Potter BJ Kang H Lee GE Bilodeau-Bertrand M Auger N. Maternal autoimmune disorders and risk of Kawasaki disease in offspring. J Pediatr. (2020) 222:240–243.e1. 10.1016/j.jpeds.2020.02.016 32171556

[32] Kawasaki T. Acute febrile mucocutaneous syndrome with lymphoid involvement with specific desquamation of the fingers and toes in children. Arerugi. (1967) 16:178–222. 6062087

[33] Sakurai Y. Autoimmune aspects of Kawasaki disease. J Investig Allergol Clin Immunol. (2019) 29:251–61. 10.18176/jiaci.0300 30183655

[34] Onouchi Y. Identification of novel Kawasaki disease susceptibility genes by genome-wide association studies. Kawasaki Dis. (2017):23–9. 10.1007/978-4-431-56039-5_4

