# Supplementary material for: The risk of pediatric cardiovascular diseases in offspring born to mothers with systemic lupus erythematosus: a nationwide study
Source: Front Pediatr. 2023 Dec 5;11:1294823. doi: 10.3389/fped.2023.1294823 (PMC10732165; doi:10.3389/fped.2023.1294823)
Supplement: Supplementary file 1 [file Table1.pdf]

Supplementary Material

Supplementary Table 1. STROBE Statement—checklist of items that should be included in reports of observational studies

	Item No	Recommendation	Page No
**Title and abstract**	1	(*a*) Indicate the study's design with a commonly used term in the title or the abstract	Title page
		(*b*) Provide in the abstract an informative and balanced summary of what was done and what was found	Introduction
**Introduction**			
Background/rationale	2	Explain the scientific background and rationale for the investigation being reported	Introduction (Paragraph 1,2)
Objectives	3	State specific objectives, including any prespecified hypotheses	Introduction (Paragraph 3)
**Methods**			
Study design	4	Present key elements of study design early in the paper	Methods (2.1)
Setting	5	Describe the setting, locations, and relevant dates, including periods of recruitment, exposure, follow-up, and data collection	Methods (2.1)
Participants	6	(*a*) *Cohort study*—Give the eligibility criteria, and the sources and methods of selection of participants. Describe methods of follow-up *Case-control study*—Give the eligibility criteria, and the sources andmethods of case ascertainment and control selection. Give the rationale for the choice of cases and controls *Cross-sectional study*—Give the eligibility criteria, and the sources and methods of selection of participants	Methods (2.1)
		(*b*) *Cohort study*—For matched studies, give matching criteria andnumber of exposed and unexposed *Case-control study*—For matched studies, give matching criteria and the number of controls per case	Methods (2.1)
Variables	7	Clearly define all outcomes, exposures, predictors, potential confounders, and effect modifiers. Give diagnostic criteria, if applicable	Methods (2.2)
Data sources/ measurement	8*	For each variable of interest, give sources of data and details of methods of assessment (measurement). Describe comparability of assessment methods if there is more than one group	Methods (2.1), Table 1
Bias	9	Describe any efforts to address potential sources of bias	Methods (2.3)
Study size	10	Explain how the study size was arrived at	Methods (2.1)
Quantitative variables	11	Explain how quantitative variables were handled in the analyses. If applicable, describe which groupings were chosen and why	Methods (2.1)
Statistical methods	12	(*a*) Describe all statistical methods, including those used to control for confounding	Methods (2.3)
		(*b*) Describe any methods used to examine subgroups and interactions	Methods (2.3)
		(*c*) Explain how missing data were addressed	Figure 1

		(*d*) *Cohort study*—If applicable, explain how loss to follow-up wasaddressed<br>*Case-control study*—If applicable, explain how matching of casesand controls was addressed<br>*Cross-sectional study*—If applicable, describe analytical methods taking account of sampling strategy	Figure 1, Table 1

		(*e*) Describe any sensitivity analyses	

Continued on next page

Results

Participants	13*	(a) Report numbers of individuals at each stage of study—eg numbers potentiallyeligible, examined for eligibility, confirmed eligible, included in the study, completing follow-up, and analysed	Figure 1, Results (Paragraph 2)
		(b) Give reasons for non-participation at each stage	Figure 1, Results (Paragraph 2)
		(c) Consider use of a flow diagram	Figure1
Descriptive data	14*	(a) Give characteristics of study participants (eg demographic, clinical, social) and information on exposures and potential confounders	Table 1, 2
		(b) Indicate number of participants with missing data for each variable of interest	Figure1
		(c) *Cohort study*—Summarise follow-up time (eg, average and total amount)	Table 1,
Outcome data	15*	*Cohort study*—Report numbers of outcome events or summary measures over time	Table 2
		Case-control study—Report numbers in each exposure category, or summary measures of exposure	
		Cross-sectional study—Report numbers of outcome events or summary measures	
Main results	16	(*a*) Give unadjusted estimates and, if applicable, confounder-adjusted estimates and their precision (eg, 95% confidence interval). Make clear which confounderswere adjusted for and why they were included	Figure 2, Results (Paragraph 3,4)
		(*b*) Report category boundaries when continuous variables were categorized	N/A
		(*c*) If relevant, consider translating estimates of relative risk into absolute risk for ameaningful time period	N/A
Other analyses	17	Report other analyses done—eg analyses of subgroups and interactions, and sensitivity analyses	N/A

Discussion

Key results	18	Summarise key results with reference to study objectives	Discussion (Paragraph 1)
Limitations	19	Discuss limitations of the study, taking into account sources of potential bias or imprecision. Discuss both direction and magnitude of any potential bias	Discussion (Paragraph 8)
Interpretation	20	Give a cautious overall interpretation of results considering objectives, limitations,multiplicity of analyses, results from similar studies, and other relevant evidence	Discussion (Paragraph 1-8)

Generalisability	21	Discuss the generalisability (external validity) of the study results	Discussion (Paragraph 3-6)
**Other information**			
Funding	22	Give the source of funding and the role of the funders for the present study and, if applicable, for the original study on which the present article is based	Funding

*Give information separately for cases and controls in case-control studies and, if applicable, for exposed and unexposed groups in cohort and cross-sectional studies.

Note: An Explanation and Elaboration article discusses each checklist item and gives methodological background and published examples of transparent reporting. The STROBE checklist is best used in conjunction with this article (freely available on the Web sites of PLoS Medicine at http://www.plosmedicine.org/, Annals of Internal Medicine at http://www.annals.org/, and Epidemiology at http://www.epidem.com/). Information on the STROBE Initiative is available at www.strobe-statement.org.

Supplementary Table 2. Description of the number of cases of congenital heart disease in children with maternal SLE group

ICD-10 codes	Disorders	Number of cases (n) *
Q202	Double outlet left ventricle	1
Q203	Discordant ventriculoarterial connection	2
Q209	Congenital malformation of cardiac chambers and connections, unspecified	1
Q210 **	Ventricular septal defect	279
Q211 ***	Atrial septal defect	882
Q212	Atrioventricular septal defect	17
Q213	Tetralogy of Fallot	7
Q218	Other congenital malformations of cardiac septa	5
Q219	Congenital malformation of cardiac septum, unspecified	16
Q220	Pulmonary valve atresia	5
Q221	Congenital pulmonary valve stenosis	40
Q222	Congenital pulmonary valve insufficiency	1
Q223	Other congenital malformations of pulmonary valve	2
Q224	Congenital tricuspid stenosis	1
Q225	Ebstein's anomaly	2

Q228	Other congenital malformations of tricuspid valve	8
Q229	Congenital malformation of tricuspid valve, unspecified	4
Q230	Congenital stenosis of aortic valve	2
Q231	Congenital insufficiency of aortic valve	5
Q233	Congenital mitral insufficiency	12
Q234	Hypoplastic left heart syndrome	1
Q238	Other congenital malformations of aortic and mitral valves	2
Q240	Dextrocardia	2
Q243	Pulmonary infundibular stenosis	2
Q244	Congenital subaortic stenosis	3
Q245	Malformation of coronary vessels	2
Q246	Congenital heart block	9
Q248	Other specified congenital malformations of heart	9
Q249	Congenital malformation of heart, unspecified	110
Q250	Patent ductus arteriosus	371
Q251	Coarctation of aorta	6
Q253	Supravalvular aortic stenosis	2

Q254	Congenital malformation of aorta	3
Q255	Atresia of pulmonary artery	5
Q256	Stenosis of pulmonary artery	46
Q257	congenital malformations of pulmonary artery	3
Q259	Congenital malformation of great arteries, unspecified	1
Q261	Persistent left superior vena cava	2
Q262	Total anomalous pulmonary venous connection	1
Q263	Partial anomalous pulmonary venous connection	1
Q265	Anomalous portal venous connection	1
Q269	Congenital malformation of great vein, unspecified	1

Abbreviations: ICD-10, World Health Organization's International Classification of Diseases, Tenth revision; SLE, Systemic lupus erythematosus. * An individual may be diagnosed with more than one congenital heart disorder. ** Includes Q210 (Ventricular septal defect), Q2100 (Muscular Ventricular septal defect), Q2101 (Perimembranous Ventricular septal defect), Q2108 (Ventricular septal defect subarterial), Q2109 (Ventricular septal defect, unspecified). *** Includes Q211 (Atrial septal defect), Q2110 (Patent or persistent foramen ovale), Q2118 (Other atrial septal defect), Q2119 (Atrial septal defect, unspecified).
