# Supplementary material for: The risk of pediatric cardiovascular diseases in offspring born to mothers with systemic lupus erythematosus: a nationwide study
Source: Front Pediatr. 2023 Dec 5;11:1294823. doi: 10.3389/fped.2023.1294823 (PMC10732165; doi:10.3389/fped.2023.1294823)
Supplement: Supplementary file 2 [file Table2.pdf]

Supplementary Material

Supplementary Table 2. Description of the number of cases of congenital heart disease in children with maternal SLE group

ICD-10 codes	Congenital heart diseases	Number of cases (n) *
Q202	Double outlet left ventricle	1
Q203	Discordant ventriculoarterial connection	2
Q209	Congenital malformation of cardiac chambers and connections, unspecified	1
Q210 **	Ventricular septal defect	279
Q211 ***	Atrial septal defect	882
Q212	Atrioventricular septal defect	17
Q213	Tetralogy of Fallot	7
Q218	Other congenital malformations of cardiac septa	5
Q219	Congenital malformation of cardiac septum, unspecified	16
Q220	Pulmonary valve atresia	5
Q221	Congenital pulmonary valve stenosis	40
Q222	Congenital pulmonary valve insufficiency	1
Q223	Other congenital malformations of pulmonary valve	2
Q224	Congenital tricuspid stenosis	1
Q225	Ebstein's anomaly	2

Q228	Other congenital malformations of tricuspid valve	8
Q229	Congenital malformation of tricuspid valve, unspecified	4
Q230	Congenital stenosis of aortic valve	2
Q231	Congenital insufficiency of aortic valve	5
Q233	Congenital mitral insufficiency	12
Q234	Hypoplastic left heart syndrome	1
Q238	Other congenital malformations of aortic and mitral valves	2
Q240	Dextrocardia	2
Q243	Pulmonary infundibular stenosis	2
Q244	Congenital subaortic stenosis	3
Q245	Malformation of coronary vessels	2
Q246	Congenital heart block	9
Q248	Other specified congenital malformations of heart	9
Q249	Congenital malformation of heart, unspecified	110
Q250	Patent ductus arteriosus	371
Q251	Coarctation of aorta	6
Q253	Supravalvular aortic stenosis	2
Q254	Congenital malformation of aorta	3
Q255	Atresia of pulmonary artery	5

Q256	Stenosis of pulmonary artery	46
Q257	congenital malformations of pulmonary artery	3
Q259	Congenital malformation of great arteries, unspecified	1
Q261	Persistent left superior vena cava	2
Q262	Total anomalous pulmonary venous connection	1
Q263	Partial anomalous pulmonary venous connection	1
Q265	Anomalous portal venous connection	1
Q269	Congenital malformation of great vein, unspecified	1

Abbreviations: ICD-10, World Health Organization's International Classification of Diseases, Tenth revision; SLE, Systemic lupus erythematosus. * An individual may be diagnosed with more than one congenital heart disorder. ** Includes Q210 (Ventricular septal defect), Q2100 (Muscular Ventricular septal defect), Q2101 (Perimembranous Ventricular septal defect), Q2108 (Ventricular septal defect subarterial), Q2109 (Ventricular septal defect, unspecified). *** Includes Q211 (Atrial septal defect), Q2110 (Patent or persistent foramen ovale), Q2118 (Other atrial septal defect), Q2119 (Atrial septal defect, unspecified).
